# 285 Development of a shared patient registry for measles exposure management amongst multiple stakeholder groups

**DOI:** 10.1017/ash.2026.10645

**Published:** 2026-06-23

**Authors:** Cindy Prins, Sri Pulaparthi, AC Burke

**Affiliations:** 1 University of Central Florida; 2 University of Central Florida College of Medicine; 3 RB Health Partners, Inc

## Abstract

**Background** Enhanced Barrier Precautions (EBP) aim to reduce multidrug-resistant organism transmission in long-term care settings. Infection preventionists play a critical role in implementing EBP, yet their experiences navigating operational challenges, staff adherence, and resident safety remain underexplored. Understanding these perspectives informs strategies for effective infection control and policy development. Methods A 42-item electronic survey was distributed via Qualtrics to 578 Florida nursing homes between May and September 2024, addressed to “Infection Control.” Ninety valid responses were received. Thirty-seven respondents provided 58 comments across seven prompts—six linked to specific survey questions and one to an open-ended prompt to share questions or thoughts about EBP. Comments were coded into nine categories and analyzed using MAXQDA Analytics Pro. Results The coding categories were: 1) Citation or Feedback, 2) Clarity and Scope of Criteria, 3) Cost and Resource Burden, 4) Effect on Infection Control Practice, 5) Effectiveness of EBP, 6) Resident Experience, 7) Staff Confusion, 8) Staffing, Workload, and Compliance, and 9) Support for EBP. Fourteen coded comments fell under Citation or Feedback, mostly related to corrective feedback or citations during F880 survey experiences, including, “Facility interpreted an action to be low contact and therefore not needing EBP while surveyor considered action to be high contact requiring full EBP”. Staffing, Workload, and Compliance had 13 coded comments, including six responses to a survey prompt asking about challenges in implementing EBP. One respondent wrote, “Non compliance in donning. No one wants to take the time to don when they are strapped for time.” Clarity and Scope of Criteria had ten coded comments, with four specifically mentioning wounds. One wrote, “I think that small skin tears should not be included into the EBP, so there is such a gray area on who should be on EBH [sic] and who doesn’t need to be. So maybe clarify skin tears.” Seven comments addressed Resident Experience, with mixed views on resident reactions. Cost and Resource Burden, Support for EBP, and Effectiveness of EBP each had four comments; Staff Confusion had two, and Effect on Infection Control Practices had one. Conclusions Infection preventionists reported significant challenges in implementing EBP, particularly staffing limitations, unclear criteria, and resource constraints. While many supported EBP’s intent, concerns about feasibility and resident impact persist. Addressing these barriers through clearer guidance, staff education, and resource allocation is essential for successful adoption and sustained infection control in long-term care settings.

